# Naphthoquinone Derivative PPE8 Induces Endoplasmic Reticulum Stress in p53 Null H1299 Cells

**DOI:** 10.1155/2015/453679

**Published:** 2015-01-18

**Authors:** Jin-Cherng Lien, Chien-Chun Huang, Te-Jung Lu, Chih-Hsiang Tseng, Ping-Jyun Sung, Hong-Zin Lee, Bo-Ying Bao, Yueh-Hsiung Kuo, Te-Ling Lu

**Affiliations:** ^1^School of Pharmacy, China Medical University, Taichung 40402, Taiwan; ^2^Department of Health and Nutrition Biotechnology, College of Medical and Health Science, Asia University, Taichung 41354, Taiwan; ^3^Department of Medical Laboratory Science and Biotechnology, Chung Hwa University of Medical Technology, Tainan 71703, Taiwan; ^4^Graduate Institute of Marine Biology, National Dong Hwa University, Pingtung 91201, Taiwan; ^5^National Museum of Marine Biology and Aquarium, Pingtung 94450, Taiwan; ^6^School of Chinese Pharmaceutical Sciences and Medicine Resources, China Medical University, Taichung 40402, Taiwan; ^7^Tsuzuki Institute for Traditional Medicine, China Medical University, Taichung 40402, Taiwan; ^8^Department of Biotechnology, Asia University, Taichung 41354, Taiwan

## Abstract

Endoplasmic reticulum (ER) plays a key role in synthesizing secretory proteins and sensing signal function in eukaryotic cells. Responding to calcium disturbance, oxidation state change, or pharmacological agents, ER transmembrane protein, inositol-regulating enzyme 1 (IRE1), senses the stress and triggers downstream signals. Glucose-regulated protein 78 (GRP78) dissociates from IRE1 to assist protein folding and guard against cell death. In prolonged ER stress, IRE1 recruits and activates apoptosis signal-regulating kinase 1 (ASK1) as well as downstream JNK for cell death. Naphthoquinones are widespread natural phenolic compounds. Vitamin K_3_, a derivative of naphthoquinone, inhibits variant tumor cell growth via oxygen uptake and oxygen stress. We synthesized a novel naphthoquinone derivative PPE8 and evaluated capacity to induce ER stress in p53 null H1299 and p53 wild-type A549 cells. In H1299 cells, PPE8 induced ER enlargement, GRP78 expression, and transient IER1 activation. Activated IRE1 recruited ASK1 for downstream JNK phosphorylation. IRE1 knockdown by siRNA attenuated PPE8-induced JNK phosphorylation and cytotoxicity. Prolonged JNK phosphorylation may be involved in PPE8-induced cytotoxicity. Such results did not arise in A549 cells, but p53 knockdown by siRNA restored PPE8-induced GRP78 expression and JNK phosphorylation. We offer a novel compound to induce ER stress and cytotoxicity in p53-deficient cancer cells, presenting an opportunity for treatment.

## 1. Introduction

ER is a central cellular organelle for newly synthesized secretory proteins and sensing signaling functions in eukaryotic cells. Alternation of oxidation state, calcium level, or pharmacological agents like tunicamycin induce accumulation of misfolded proteins. To restore favorable folding environment, ER membrane expands massively, which may tolerate more misfolded proteins and promote their folding [1]. Also, ER transmembrane protein, IRE1, senses ER stress and is phosphorylated to induce ER stress response genes [2]. Chaperone protein GRP78 dissociates from IRE1 to assist protein folding and protect against cell death [3]. If cells fail to restore folding capacity, IRE1 pathway contributes to apoptosis. IRE1 reportedly recruits ASK1, a member of mitogen-activated protein kinase (MAP3K), activating c-Jun N-terminal kinase (JNK) and p38 pathways [4]. Phosphorylated JNK translocates to nuclei to phosphorylate and transactivate c-Jun that is involved in transcription of various proteins, some known as proapoptotic [3, 5].

JNK also phosphorylates p53, a transcription factor, promoting p53-mediated apoptosis to prevent cell transformation. Loss of p53 is the most common genetic alternation in cancer. Early preclinical study proved that tumors with wild-type p53 are more sensitive to chemoradiation [6]. Activation of p53 is linked with apoptosis, but accumulating evidence indicates that p53 regulates prosurvival genes, depending on growth environment, type of stress, and cellular context; for example, p53 protects cells against UV-induced apoptosis by binding and inactivating JNK [7]. Concanavalin A, a carbohydrate-binding protein extracted from jack beans, induces p53-deficient cell apoptosis; however, rescue of p53 function in the same cells protects them by inducing G1 arrest [8]. Metformin, a diabetic drug, selectively inhibits p53-deficient tumor cell transformation by activating AMPK and inhibiting oxidative phosphorylation, rendering an environment more vulnerable to p53-deficient tumor cells [9]. The cells lacking functional p53 may become more vulnerable in response to some agents, which could be an alternative strategy for cancer therapy.

**Figure 1:**
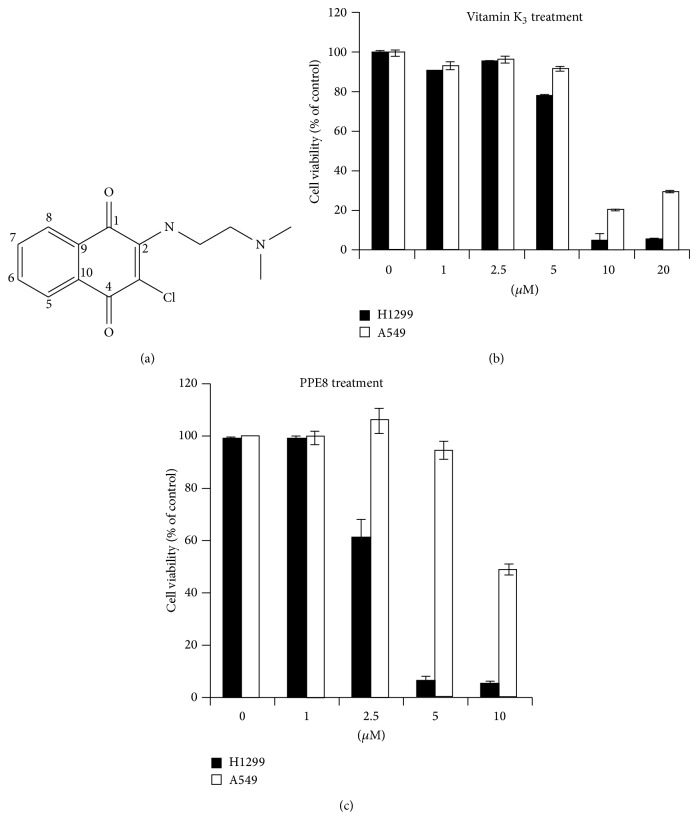
(a) Structures of PPE8, (b) A549, and H1299 cells were incubated without or with indicated concentrations of vitamin K_3_ or (c) PPE8 for 24 h. The cell viability was examined by MTS assay and expressed relative to viability of the untreated cells (100% control value). Similar results were obtained in three independent experiments. Results were expressed as the mean ± SD.

Naphthoquinones, secondary metabolites widespread in nature, serve as organic dyes [10]. Their derivatives have biological activities, for example, antitumor, antibacterial, anti-inflammatory, antiparasitic, and cytotoxic activities. For example, menadione (2-methyl-naphthoquinone), a synthetic chemical compound, serves as nutritional supplement due to its vitamin K_3_ activity. In addition, vitamin K_3_ reportedly causes oxygen uptake and oxygen stress by interaction with reduced glutathione [11]. The reactive oxygen species (ROS) generation by vitamin K_3_ causes pancreatic cell apoptosis [12]. Other vitamin K analogs with* S*- or* O*-ethers at position 3 of quinoid nuclei reportedly generate ROS and interact with cellular thiols, thus changing cellular oxygen state. These analogs have more potential to inhibit hepatoma cell growth [13]. We investigated whether vitamin K_3_ could inhibit lung cancer cell growth. The 50% inhibitory concentration was more than 5 *μ*M in H1299 and A549 cells (Figure 1(b)). These findings prompted us to synthesize more potent naphthoquinone derivatives to inhibit lung cancer cell growth. PPE8, one of derivatives, has the naphthoquinone structure with nitrogen link at position 2 and chlorine at position 3 of quinoid nuclei (Figure 1(a)). Interestingly, we found that H1299 cells are more sensitive to PPE8 than A549 cells. Stress markers p-IRE1, GRP78, and p-JNK were examined by western blot, ER was confirmed by ER-specific dye immunostain, and fluorescent area was analyzed by ImageJ software. Whether p53 was involved in PPE8-induced ER stress was investigated by p53 siRNA knockdown in A549 cells.

## 2. Materials and Methods

### 2.1. PPE8 Synthesis, Reagents, and Antibodies

PPE8 [3-chloro-2-(*N*,*N*-dimethylaminoethylamino)-l,4-naphthoquinone] was synthesized as follows. Suspension of 2,3-dichloro-1,4-naphthoquinone (5 g, 0.02 mol) in benzene was added to excess of* N*,*N*-dimethylethylenediamine. The reaction mixture was stirred for 30 min at room temperature and filtered. Precipitate was recrystallized from ethanol to yield 5 g (93%) of dark-red needle PPE8 crystals. Melting point was 73-74°C. EIMS (*m/z*): 277 (M^+^). ^1^H NMR (CDCl_3_, 200 MHz) (ppm): 2.29 (6H,* s*, –N(CH_3_)_2_), 2.58 (2H,* t*,* J* = 6.0 Hz, –CH_2_N=), 3.89–3.95 (2H,* m*, –NHCH_2_–), 6.02 (1H,* br*., –NH–), 7.58–7.74 (2H,* m*, H-6, 7), 8.00–8.03 (1H,* m*, H-5), and 8.13–8.16 (1H,* m*, H-8). Phenylmethylsulfonyl fluoride (PMSF), RNaseA, saponin, and triton X-100 were obtained from Sigma (St. Louis, MO); ER-specific dye was obtained from Chemicon (Billerica, MA); antibodies for p53 and *β*-actin were obtained from Santa Cruz Biotechnology (Santa Cruz, CA); antibodies for IRE1, GRP78, JNK, p-JNK, and ASK1 were obtained from Cell Signaling (Danvers, MA). Antibody for p-IRE1 at serine 724 was obtained from GeneTex, Inc. (North America); anti-rabbit and anti-mouse IgG-horseradish peroxidases were obtained from Jackson Immunoresearch (West Grove, PA).

### 2.2. Cell Culture

A549 and H1299 cells obtained from Bioresource Collection and Research Center were cultured in DMEM medium with 10% fetal bovine serum (FBS) (HyClone Laboratories, Logan, UT) and then incubated at 37°C with 5% CO_2_.

### 2.3. MTS Assay

Cell viability was examined by using the CellTiter 96 aqueous one-solution cell proliferation kit (Promega, Madison, WI). Approximately 4∗10^3^ cells were seeded in each 96-well plate and allowed to adhere overnight. Cells were treated with various concentrations of PPE8 for 24 h and then were replaced with 100 *μ*L medium containing 20 *μ*L MTS at 37°C for 1 h. Absorbance was measured at 490 nm with a SpectraMax M2 microplate reader (Molecular Devices, Menlo Park, CA). The percentage of cell viability was calculated as follows: (absorbance of treated cultures−absorbance of background control)/(absorbance of control cultures−absorbance of background).

### 2.4. ER Stain

Approximately 1∗10^5^ cells on coverslips were treated with or without PPE8, fixed with 4% paraformaldehyde at 4°C for 10 min, permeabilized with 0.5% saponin in phosphate buffered saline (PBS) for 20 min, and stained with 0.3 *μ*g/mL of ER-specific dye and 0.5 *μ*g/mL DAPI. Slides were mounted using a SlowFade antifade kit (Molecular Probes, Eugene) and examined using a Nikon Eclipse TE2000-S microscope with a 20x Plan Fluor objective and a UV filter. The final composite image was created using Adobe Photoshop 7.0. The total ER area (green) of each field was measured and summed by ImageJ. The average of ER area of each cell was calculated by dividing numbers of cells. At least 100 cells were measured in each experiment.

### 2.5. Western Blot Analysis

Cells were lysed with lysis buffer containing 50 mM Tris pH 7.2, 150 mM NaCl, 1 mM EDTA, and 1% triton X-100 containing protease inhibitors (1 mM PMSF, 10 *μ*g/mL aprotinin, and 1 mM Na_3_VO_4_). For detection p-IRE1, SDS was added to lysis buffer to a final concentration of 0.5%. The protein concentration was assayed by the BCA protein assay kit (Pierce, Rockford, IL). Equal amount of protein was resolved by sodium dodecyl sulfate-polyacrylamide gel electrophoresis (SDS-PAGE) and transferred to a polyvinylidene difluoride membrane. Membranes were immunoblotted with primary antibodies and horseradish peroxidase-conjugated secondary antibodies. Protein signals were visualized by an enhanced chemiluminescence assay (Amersham, Piscataway, NJ). The chemiluminescence photographs were taken with LAS-4000 Image Analyzer (Fujifilm, Japan) and processed by Adobe Photoshop 7.0 (Adobe System Inc., San Jose, CA).

### 2.6. Immunoprecipitation

H1299 cells (1∗10^6^ cells) were cultured overnight and then treated with or without PPE8. Cell lysates were collected with lysis buffer. The protein concentration was assayed using the BCA protein assay kit. For immunoprecipitation of ASK1, the catch and release reversible immunoprecipitation system (Upstate, Temecula, CA) was employed. Briefly, 500 *μ*g of cell lysates was incubated with 2 *μ*g of ASK1 antibody and affinity ligand overnight at 4°C. Unbound proteins were washed away with wash buffer. The bound ASK1 was eluted by 100 *μ*L denaturing buffer containing 5% *β*-mercaptoethanol at 90°C for 10 min. The supernatant was centrifuged and equal amount of supernatant was resolved on SDS-PAGE for western blot analysis.

### 2.7. Plasmid, Small Interfering RNA (siRNA), and Transfection

Mammalian p53 siRNA expression plasmid was kindly provided by Dr. M.-D. Lai from the Graduate Institute of Clinical Medicine at National Cheng Kung University, Tainan, Taiwan. The siRNA purchased from Santa Cruz Biotechnology contains a pool of 4 target-specific 20–25 nucleotide siRNAs against respective target genes. The sequences for siRNA duplexes targeting IRE1 were sense CAACCUCUCUUCUGUAUCUtt, GGAAGGUGAUGCACAUCAAtt, CUGGAGGAGACGAAUGAUAtt, and CUGUACUCUUGGAGUAACAtt; antisense AGAUACAGAAGAGAGGUUGtt, GGAAGGUGAUGCACAUCAAtt, CUGGAGGAGACGAAUGAUAtt, and CUGUACUCUUGGAGUAACAtt. The sequences for nontargeting siRNA were sense UUCUCCGAACGUGUCACGUtt and antisense ACGUGACACGUUCGGAGAAtt. A549 or H1299 cells at 50% confluence in six-well dishes were transfected with 60 pmol of siRNA and 10 *μ*L Lipofectamine 2000 (Invitrogen) for siRNA transfection; 1.2 *μ*g of p53 plasmid and 8 *μ*L Lipofectamine 2000 were used for p53 transfection. After incubation for 24 h, cells were treated without or with PPE8. The cell lysates were collected with lysis buffer and then processed by western blot.

## 3. Results

### 3.1. PPE8 Inhibits Lung Cancer Cell Viability

We probed effect of vitamin K_3_ and PPE8 on lung cancer cell viability (A549 and H1299 cells). A549 cells are p53 normal and H1299 cells are p53 null. After 24-hour vitamin K_3_ treatment, 50% inhibitory concentration (IC_50_) values were more than 5 *μ*M for A549 and H1299 cells (Figure 1(b)). After 24-hour PPE8 treatment, IC_50_ values were about 10 *μ*M for A549 and 3.2 *μ*M for H1299 cells (Figure 1(c)). These results showed that PPE8 is 3 times more potent to H1299 cells than A549 cells.

### 3.2. PPE8 Induces ER Stress

**Figure 2:**
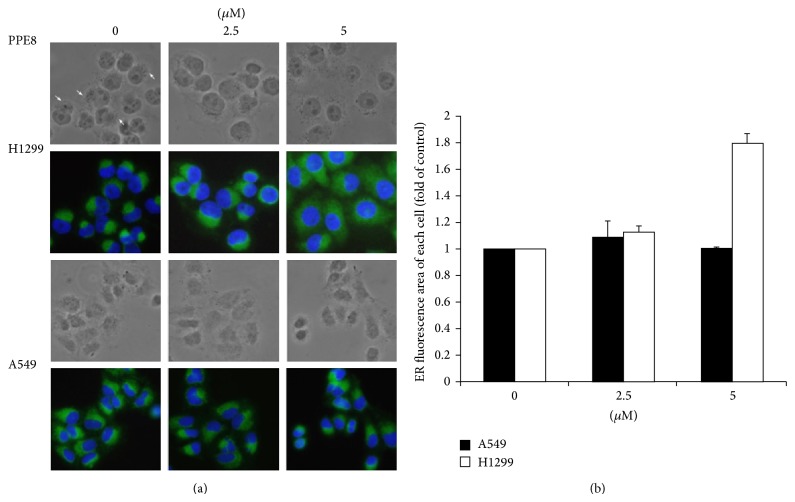
(a) A549 and H1299 cells were seeded on coverslips overnight. The cells were treated without or with indicated concentrations of PPE8 for 4 h. Cells were fixed and immunostained with ER-specific dye (green) and DAPI (blue). After 5 *μ*M PPE8 treatment, the structures near nuclei could not be observed compared with control cells (arrows) in bright-field microscope. (b) The average green area of each cell was measured (described in methods) without or with indicated concentrations of PPE8 treatment. The average of ER area in cells without PPE8 treatment was expressed as control. Bars represented mean ± SD and expressed as a fold increase from control. Similar results were obtained in three independent experiments.

To test killing effect, morphology was examined at various PPE8 concentrations. Following 4-hour PPE8 treatment, the characteristic features of apoptosis, including chromatin condensation and apoptotic body, were not observed by DAPI staining (blue) in H1299 and A549 cells (Figure 2(a)). However, the structure located near nuclei was not observed after 5 *μ*M PPE8 treatment compared with control cells in bright-field microscope (Figure 2(a), arrows). To further investigate the structures, ER specific immunofluorescent dyes (green) were used. We found that H1299 cells had expanded ER following 5 *μ*M PPE8 treatment (Figure 2(a), upper green panel). Analyzing area of green fluorescence by ImageJ indicated that PPE8 induced 1.5-fold increase over cells untreated (Figure 2(b)). Still, A549 cells showed no significant difference in ER morphological change (Figure 2(a), lower panel, and Figure 2(b)). Thus, these results indicated that PPE8 induced ER expansion in H1299 cells.

**Figure 3:**
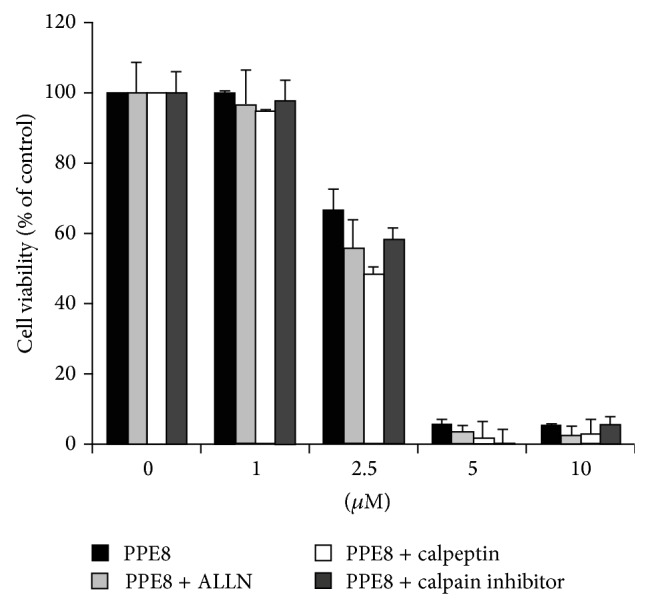
H1299 cells were incubated without or with 5 *μ*M ALLN, 10 *μ*M calpeptin, or 10 *μ*M calpain inhibitor in combination with 5 *μ*M PPE8 for 24 h; the cell viability was examined by MTS assay and expressed relative to viability of cells without PPE8 treatment (100% control value). Similar results were obtained in three independent experiments. Results were expressed as the mean ± SD.

ER is an organelle involved in control of cell activities through calcium signaling. Disturbances in calcium regulation lead to calcium release, which in turn activates calpain. The activated calpain cleaves downstream caspase cascade, causing cell death [14]. To evaluate whether calpain was involved in PPE8-induced cell death, calpain inhibitors, including ALLN, calpeptin, and calpain inhibitor, were incubated with PPE8-treated H1299 cells. These inhibitors failed to prevent PPE8-induced cell death (Figure 3). In addition, we could not find caspase 3 activation (data not shown), suggesting calpain and downstream caspases were not involved in PPE8-induced cell death.

### 3.3. IRE1 Is Involved in PPE8-Induced ER Stress

**Figure 4:**
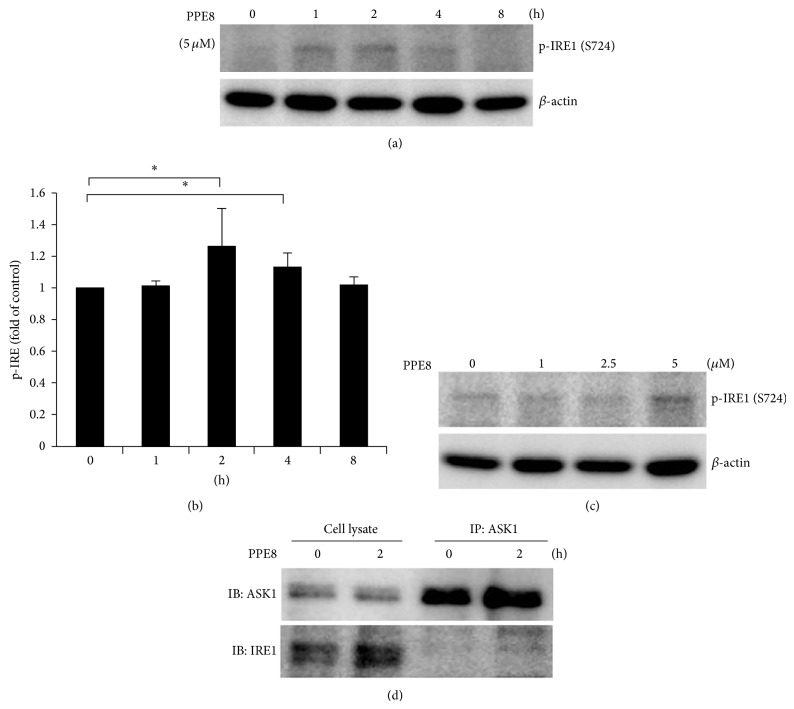
H1299 cells were treated without or with (a) 5 *μ*M PPE8 for indicated time. (b) Quantification of p-IRE of 4 independent experiments. The signal detected in untreated cells was taken as control of each experiment. Bars represented mean ± SD and expressed as a fold increase from control.* Asterisks* indicate the values that are significantly different from corresponding control (*P* < 0.05). (c) H1299 cells were treated with indicated concentrations of PPE8 for 2 h. The cell lysates were processed for western blotting to examine phosphorylated IRE1 with p-IRE1 antibody. (d) H1299 cells were treated without or with 5 *μ*M PPE8 for 2 h. The lysates were immunoprecipitated with ASK1 antibody. The eluted protein was processed for western blotting to examine IRE1 with IRE1 antibody. Similar results were obtained in three independent experiments.

In response to ER stress, IRE1 is activated and phosphorylated at serine 724 [15]. To investigate whether IRE1 was activated after PPE8 treatment, we used p-IRE1 (S724) antibody to gauge IRE1 phosphorylation. After 1-2 h of exposure to 5 *μ*M PPE8, IRE1 phosphorylation level was prominent and then attenuated with 8-hour PPE8 exposure, indicating PPE8-induced IRE phosphorylation was transient (Figure 4(a)). With 1, 2.5, and 5 *μ*M PPE8 treatment for 2 h, 5 *μ*M PPE8 induced IRE1 phosphorylation (Figure 4(c)). These results indicated that 5 *μ*M PPE8 induced transient IRE1 phosphorylation during PPE8 treatment for 1–4 h. Upon activation, IRE1 recruits ASK1, resulting in JNK activation, which is implicated in ER stress-induced cell death [4]. To investigate whether PPE8 induced IRE1-ASK1 complex formation, ASK1 was immunoprecipitated and coimmunoprecipitated IRE1 was then detected.  Figure 4(d) showed that ASK1 was immunoprecipitated in cells with or without PPE8 treatment; however, more IRE1 was coimmunoprecipitated with ASK1 in cells following PPE8 treatment (Figure 4(d), lane 4 compared with lane 3). These results indicated that PPE8 induced IRE1-ASK1 complex formation.

### 3.4. JNK Is Activated after PPE8 Treatment

**Figure 5:**
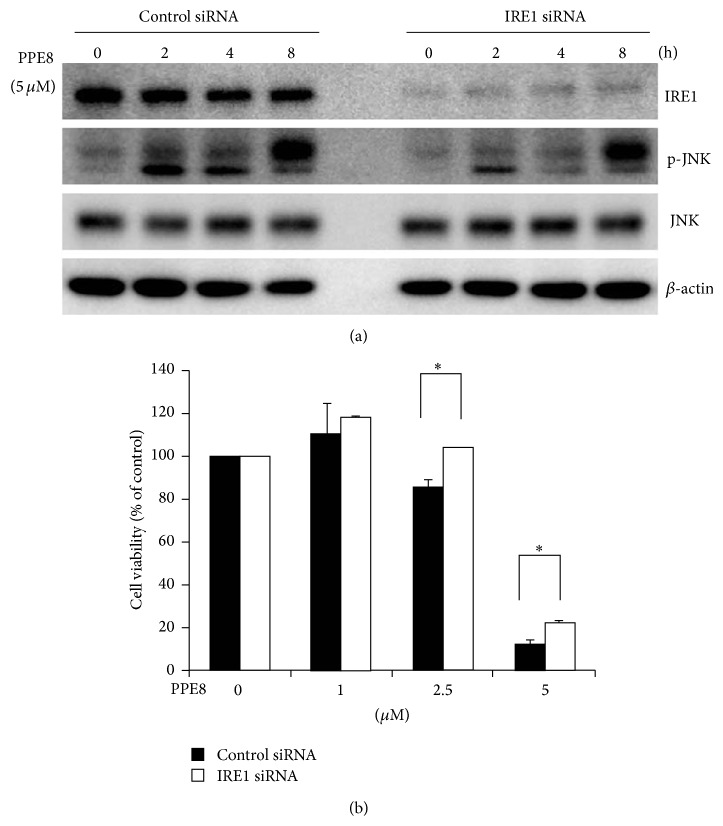
H1299 cells were transfected with control siRNA or IRE1 siRNA for 24 h. (a) The cells were then treated with 5 *μ*M PPE8 for indicated time. The cell lysates were immunoblotted with IRE1, JNK, p-JNK, and *β*-actin antibodies. (b) The cells were then treated with indicated concentrations of PPE8 for 12 h. The cell viability was measured by MTS assay and expressed relative to viability of the cells without PPE8 treatment (100% control value). Similar results were obtained in three independent experiments. Results were expressed as the mean ± SD.* Asterisks* indicate the values that are significantly different from corresponding control siRNA (*P* < 0.05).

To ascertain whether interaction of IRE1 and ASK1 leads to JNK activation, phosphorylated JNK was examined after PPE8 treatment for 2 to 8 h. We found that JNK was phosphorylated afterwards (Figure 5(a), left panel), but IRE1 knockdown by siRNA only partially attenuated JNK phosphorylation following 4-hour PPE8 treatment. However, this attenuated JNK phosphorylation could not sustain for 8-hour PPE8 treatment in IRE1 knockdown cells (Figure 5(a), right panel). Results imply signals other than IRE1 inducing JNK activation after 8-hour PPE8 treatment. Next, we investigated whether IRE1 knockdown could rescue PPE8-induced cell death. We found that PPE8-induced cell death was partially rescued in IRE1 knockdown cells after 2.5 and 5 *μ*M PPE8 treatment for 12 h (Figure 5(b)). Taken together, PPE8 induced ER stress and IRE1-ASK1 complex formation, leading to downstream JNK activation and H1299 cell death.

### 3.5. p53 Null Cells Are More Sensitive to PPE8-Induced ER Stress

**Figure 6:**
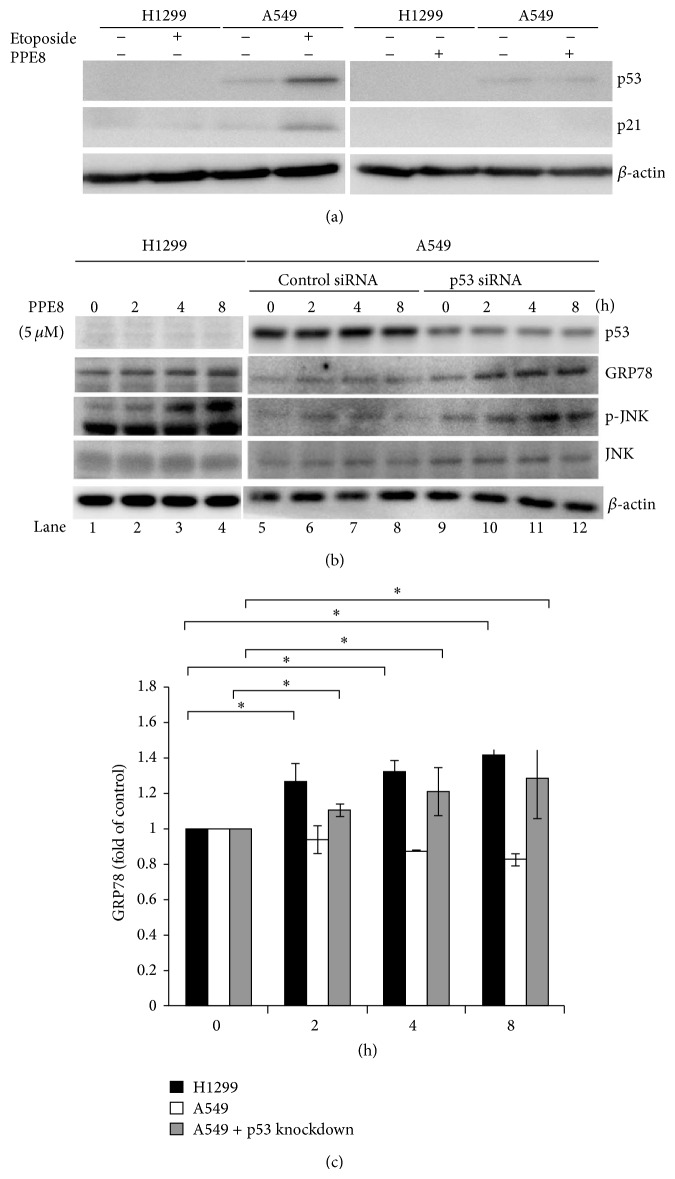
(a) H1299 and A549 cells were treated without or with etoposide (100 *μ*g/mL) or 5 *μ*M PPE8 treatment for 8 h. (b) Before PPE8 treatment, A549 cells were transfected with control siRNA or p53 siRNA for 24 h. The cell lysates were collected from H1299 and A549 cells and immunoblotted with p53, p21, GRP78, p-JNK, JNK, and *β*-actin antibodies. (c) Quantification of GRP78 of 3 independent experiments. The signal detected in untreated cells was taken as control of each experiment. Bars represented mean ± SD and expressed as a fold increase from control.* Asterisks* indicate the values that are significantly different from corresponding control (*P* < 0.05).

Tumor suppressor protein p53 plays a pivotal role in controlling cell cycle, DNA repair, and apoptosis in response to various stresses. It is reported that p53 is involved in etoposide-induced ER stress [16]; therefore, we examined p53 role in PPE8-treated cells. Induction of p53 and its target gene p21 was detected by etoposide in A549 cells but not in H1299 cells. However, PPE8 could not stimulate p53 and p21 induction in both A549 and H1299 cells (Figure 6(a)). These results indicated that p53 and p21 activation was not involved in PPE8-induced cytotoxicity. On the other hand, p53 function is resistant to cytotoxicity via cell cycle arrest which protects cell from death. Tunicamycin administration induces GRP78 expression more potently in p53-deficient mice than in controls, indicating p53 protects cells from hepatotoxic effects in chronic ER stress [17]. To determine whether downregulation of p53 was required for PPE8-induced ER stress, p53 was knocked down by siRNA in A549 cells. GRP78 expression and JNK phosphorylation were examined in p53 downregulation cells. As Figure 6(b) showed, p53 was expressed in A549 but not in H1299 cells. With PPE8 treatment for 2–8 h, JNK was phosphorylated and GRP78 expression increased in H1299 cells (Figure 6(b), lanes 1–4); however, PPE8 failed to induce JNK phosphorylation and GRP78 expression in A549 cells (Figure 6(b), lanes 5–8). Reducing p53 expression by siRNA in A549 cells increased both JNK phosphorylation and GRP78 expression following PPE8 treatment for 2–8 h (Figure 6(b), lanes 9–12). These results indicated that PPE8-induced ER stress and cell death were more potent in p53 null cells than in p53 normal cells.

## 4. Discussion

Our study showed that naphthoquinone derivative PPE8 induced ER stress in p53-null H1299 cells. PPE8 induced ER stress via ER expansion, GRP78 expression, and IRE1 activation, which in turn recruited ASK1 and downstream JNK phosphorylation. However, PPE8 could not induce ER expansion, JNK phosphorylation, and GRP78 expression in A549 cells. Interestingly, PPE8-induced GRP78 expression and JNK activation were partially restored in p53 knockdown A549 cells. Data indicate PPE8 as ER stress inducer in cells lacking p53, which could be a candidate to treat p53 null lung cancer.

The oxidative environment of the ER is important for protein folding by formation of disulfide bonds between two cysteine residues in proteins. Exposure of cells to oxidation state change causes unfolded protein accumulation in ER. Several naphthoquinone derivatives reportedly induce oxidation state change. Menadione causes oxygen uptake and oxygen stress by interaction of menadione with glutathione [11]. Diosquinone inhibited lipopolysaccharide-induced NO production by reduction of superoxide radical [18]. Vitamin K analogs with* S*- or* O*-ethers at position 3 of quinoid nuclei have more potential to inhibit cell growth [13]; they are reported as changing oxidation state in cells. To synthesize more potent growth inhibitory structures, we substituted position 2 of quinoid nuclei with nitrogen linked aliphatic side chains, PPE8. Whether PPE8-induced ER stress especially in p53-deficient H1299 cells is involved in oxidation state change needs further investigation.

The ER is a large and highly dynamic organelle. Cells constantly adjust ER sizes and shapes according to need. ER enlargement through increase of membrane amount is needed to accommodate more proteins under ER stress. Ino2/4 is activated in regulating amount of ER membrane through stimulation of lipid biosynthesis [1]. In addition, microtubules are involved in regulating ER morphology, trafficking, and expansion. GRP78 interacts with *β*III-tubulin, but the functional consequences of this association are unknown [19]. Nonmuscle myosin II (NMII) is required for IRE1 oligomerization and signaling [20]. Its downstream XBP1 activation is also involved in regulating amount of ER membrane in fibroblasts and B lymphocytes [21]. These observations suggest a link between the microtubule cytoskeleton and the initiation of ER stress responses. Under fluorescence microscopy observation, PPE8-induced ER enlargement was confirmed by ER-specific dye (Figure 2). In addition, PPE8 induced IRE1 and GRP78 activation in H1299 cells. These results implied that PPE8 may regulate cytoskeleton and downstream signaling.

In response to ER stress, both PERK and IRE1 sense unfolded proteins in ER lumen and transduce signal to downstream molecules. Lin et al. reported that chronic ER stress led to IRE1 inactivation; however, PERK signaling was unaffected. Termination of IRE1 activity is a major factor in allowing cell death after unfolded protein activation [22]. Figure 4(a) showed that IRE1 was activated only after 5 *μ*M PPE8 treatment for 2–4 h, indicating that PPE8-induced IRE1 activation was terminated after PPE8 treatment for 4 h. These results implied that PPE8-induced transient IRE1 activation may not continually restore favorable environment and subsequently induce cell death in H1299 cells.

Both IRE1 and PERK are involved in JNK phosphorylation and cell death [4]. Knockdown PERK attenuated tunicamycin-induced JNK phosphorylation, implying a PERK role in ER stress-induced JNK phosphorylation [23]. We showed that IRE1 was activated only after 5 *μ*M PPE8 treatment for 2–4 h; however, JNK phosphorylation persisted to 8 h (Figure 5(a), left panel). In IRE1 knockdown cells, PPE8-induced JNK phosphorylation was attenuated by 2–4 h; JNK was still activated after PPE8 treatment for 8 h (Figure 5(a), right panel). These results implied that PERK or other molecules might be involved in long-term, PPE8-induced JNK phosphorylation.

p53 and its target gene p21 play a key role in apoptosis responding to DNA damage. Some strategies try to restore its function to suppress cell growth and tumor size, when combined with therapeutic agents [5]. Conversely, p53 and p21 function spawns resistance to cytotoxicity via cell cycle arrest to exert cytoprotective effect against cell death. It is reported that high levels of p21 induce cell cycle arrest to facilitate the prosurvival role during tunicamycin treatment [24]. PPE8 could not induce p21 expression in A549 cells, indicating that resistance of A549 cells to PPE8 was not due to p21 induction. In addition, PPE8 could not change p21 level in H1299 cells (Figure 6(a)), indicating that p21 may not be involved in PPE8-mediated cytotoxicity. On the other hand, tunicamycin administration induces GRP78 expression more potently in p53-deficient mice than in controls, indicating p53 protects cells from hepatotoxic effects in chronic ER stress [17]. Concanavalin A induces p53-null ovarian cancer apoptosis. Isogenic pairs, SKP53 and TR9-7 expressing wild-type p53, were far less sensitive to concanavalin A [8]. Although we could not detect p21 expression in H1299 cells (Figure 6(a)), we proved that PPE8 was less cytotoxic to A549 cells that express wild type p53. PPE8-induced ER stress was partially restored to induce JNK phosphorylation and GRP78 expression in A549 cells with p53 knockdown (Figure 6(b)). These results indicated that PPE8 renders an environment more vulnerable to p53-deficient tumor cells. We devised new naphthoquinone derivative PPE8, exerting cytotoxicity on cells lacking p53.

## Abbreviation

ASK1: Apoptosis signal-regulating kinase 1
ER: Endoplasmic reticulum
GRP78: Glucose-regulated protein 78
IRE1: Inositol-regulating enzyme 1.
